# Supplementary material for: Developing adapted wheat lines with broad-spectrum resistance to stem rust: Introgression of Sr59 through backcrossing and selections based on genotyping-by-sequencing data
Source: PLoS One. 2023 Oct 12;18(10):e0292724. doi: 10.1371/journal.pone.0292724 (PMC10569509; doi:10.1371/journal.pone.0292724)
Supplement: Attachment — Submitted filename: PONE-D-23-21095.pdf [file pone.0292724.s001.pdf]

PLOS ONE

Developing adapted wheat lines with broad-spectrum resistance against stem rust: Introgression of Sr59 through backcrossing and selections based on genotyping-by-sequencing data

--Manuscript Draft--

Manuscript Number:	PONE-D-23-21095
Article Type:	Research Article
Full Title:	Developing adapted wheat lines with broad-spectrum resistance against stem rust: Introgression of Sr59 through backcrossing and selections based on genotyping-by-sequencing data
Short Title:	Enhancing Stem Rust Resistance in Wheat
Corresponding Author:	Mahbubjon Rahmatov, Ph.D.<br>Swedish University of Agricultural Sciences: Sveriges lantbruksuniversitet<br>Alnarp, SWEDEN
Keywords:	KASP markers, Plant Breeding, Puccinia graminis, Secale cereale, Translocation, Triticum aestivum
Abstract:	Wheat breeding against stem rust, caused by Puccinia graminis Pers. f.sp. tritici Eriks. and E. Henn. (Pgt), a highly devastating fungal disease of bread wheat (Triticum aestivum L.), is constantly challenged by the frequent emergence of new virulent Pgt races across wheat-growing continents, posing significant obstacles to resistance breeding in wheat. Utilizing combinations of broad-spectrum resistance genes would contribute to durable resistance against stem rust disease. The present study aimed at transferring a newly defined stem rust resistance gene, Sr59 (from line TA5094). This line was developed through a CSph1bM-induced T2DS·2RL Robertsonian translocation conferring broad-spectrum resistance. However, due to the poor agronomic performance of line TA5094, it was necessary to eliminate the CSph1bM background to transfer Sr59 into more adapted genetic backgrounds and assess its utility in wheat improvement. A total of 2070 BC1F1 and 1230 BC2F1 plants were derived from backcrossing of BAJ#1, KACHU#1, and REEDLING#1 with TA5094, as determined through combined stem rust seedling assays and molecular marker analyses. Genotyping-by-sequencing (GBS) results revealed the physical positions of 15,116 SNPs on chromosome 2R. Results showed that the adapted wheat genotypes used for backcrossing did not possess broad-spectrum resistance to the stem rust races tested, in contrast to line TA5094, which contains Sr59 and shows resistance to all tested races. The combined approach of stem rust seedling assays and kompetitive allele specific PCR (KASP) marker analysis proved successful in selecting and generating later generations, such as the BC2F2 population, that contained the Sr59 gene - a finding also confirmed by GBS. In early-generation data from backcrossing, however, segregations diverged from 3:1, indicating that more than one gene may be responsible for the resistance reactions of Sr59. The use of GBS markers data (based on 40,584 SNPs across all seven wheat chromosomes) to transfer the recurrent parent background from the adapted cultivars to the later generation populations resulted in an average genome recovery of 71.2% in BAJ#1*2/TA5094, 69.8% in KACHU#1*2/TA5094, and 70.5% in REEDLING#1*2/TA5094 backcrossing populations. The GBS data verified a stable introgression of Sr59 in the BC2F2 populations, evidenced by the presence of the Ph1 locus and the absence of the 50,936,209 bp deletion, spanning from 396,630,846 bp to 447,567,055 bp, which is characteristic of the CSph1bM at the Ph1 locus. Selection across generations up to BC2F9, using a combination of phenotypic selections, stem rust seedling assays, KASP markers, and GBS data, substantially accelerated the transfer of broad-spectrum resistance into adapted wheat genotypes, thus creating a form of speed breeding. In conclusion, this study demonstrates the successful introduction of the Sr59 resistance gene into elite genetic backgrounds, providing a significant resource for wheat improvement to mitigate yield losses caused by stem rust.
Order of Authors:	Mahbubjon Rahmatov, Ph.D.
	Mahboobeh Yazdani

Powered by Editorial Manager® and ProduXion Manager® from Aries Systems Corporation

	Matthew N. Rouse
	Brian J. Steffenson
	Prabin Bajgain
	Mehran Patpour
	Eva Johansson

Additional Information:	
**Question**	**Response**
**Financial Disclosure**<br><br>Enter a financial disclosure statement that describes the sources of funding for the work included in this submission. Review the submission guidelines for detailed requirements. View published research articles from *PLOS ONE* for specific examples.<br><br>This statement is required for submission and **will appear in the published article** if the submission is accepted. Please make sure it is accurate.<br><br>**Unfunded studies**<br>Enter: *The author(s) received no specific funding for this work.*<br><br>**Funded studies**<br>Enter a statement with the following details:<br>• Initials of the authors who received each award<br>• Grant numbers awarded to each author<br>• The full name of each funder<br>• URL of each funder website<br>• Did the sponsors or funders play any role in the study design, data collection and analysis, decision to publish, or preparation of the manuscript?<br>• **NO** - Include this sentence at the end of your statement: *The funders had no role in study design, data collection and analysis, decision to publish, or preparation of the manuscript.*<br>• **YES** - Specify the role(s) played.<br><br>* typeset	For this research financial support obtained from the Swedish Research Council Vetenskapsrådet and FORMAS
**Competing Interests**<br><br>Use the instructions below to enter a competing interest statement for this submission. On behalf of all authors,	There are no competing interests among the author.

Powered by Editorial Manager® and ProduXion Manager® from Aries Systems Corporation

disclose any competing interests that could be perceived to bias this work—acknowledging all financial support and any other relevant financial or non-financial competing interests.<br><br>This statement is required for submission and **will appear in the published article** if the submission is accepted. Please make sure it is accurate and that any funding sources listed in your Funding Information later in the submission form are also declared in your Financial Disclosure statement.<br><br>View published research articles from *PLOS ONE* for specific examples.<br><br>**NO authors have competing interests**<br><br>Enter: *The authors have declared that no competing interests exist.*<br><br>**Authors with competing interests**<br><br>Enter competing interest details beginning with this statement:<br><br>*I have read the journal's policy and the authors of this manuscript have the following competing interests: [insert competing interests here]*<br><br>* typeset	
**Ethics Statement**<br><br>Enter an ethics statement for this submission. This statement is required if the study involved:<br><br>• Human participants<br>• Human specimens or tissue<br>• Vertebrate animals or cephalopods<br>• Vertebrate embryos or tissues<br>• Field research<br><br>Write "N/A" if the submission does not require an ethics statement.	There is no any ethical issues in the study.

General guidance is provided below. Consult the submission guidelines for detailed instructions. **Make sure that all information entered here is included in the Methods section of the manuscript.**<br><br>**Format for specific study types**<br><br>**Human Subject Research (involving human participants and/or tissue)**<br>• Give the name of the institutional review board or ethics committee that approved the study<br>• Include the approval number and/or a statement indicating approval of this research<br>• Indicate the form of consent obtained (written/oral) or the reason that consent was not obtained (e.g. the data were analyzed anonymously)<br><br>**Animal Research (involving vertebrate animals, embryos or tissues)**<br>• Provide the name of the Institutional Animal Care and Use Committee (IACUC) or other relevant ethics board that reviewed the study protocol, and indicate whether they approved this research or granted a formal waiver of ethical approval<br>• Include an approval number if one was obtained<br>• If the study involved *non-human primates*, add *additional details* about animal welfare and steps taken to ameliorate suffering<br>• If anesthesia, euthanasia, or any kind of animal sacrifice is part of the study, include briefly which substances and/or methods were applied<br><br>**Field Research**<br><br>Include the following details if this study involves the collection of plant, animal, or other materials from a natural setting:<br>• Field permit number<br>• Name of the institution or relevant body that granted permission	
**Data Availability**<br><br>Authors are required to make all data	Yes - all data are fully available without restriction

Powered by Editorial Manager® and ProduXion Manager® from Aries Systems Corporation

underlying the findings described fully available, without restriction, and from the time of publication. PLOS allows rare exceptions to address legal and ethical concerns. See the PLOS Data Policy and FAQ for detailed information.<br><br>A Data Availability Statement describing where the data can be found is required at submission. Your answers to this question constitute the Data Availability Statement and **will be published in the article**, if accepted.<br><br>**Important:** Stating 'data available on request from the author' is not sufficient. If your data are only available upon request, select 'No' for the first question and explain your exceptional situation in the text box.<br><br>Do the authors confirm that all data underlying the findings described in their manuscript are fully available without restriction?	
**Describe where the data may be found in full sentences. If you are copying our sample text, replace any instances of XXX with the appropriate details.**<br><br>• If the data are **held or will be held in a public repository**, include URLs, accession numbers or DOIs. If this information will only be available after acceptance, indicate this by ticking the box below. For example: *All XXX files are available from the XXX database (accession number(s) XXX, XXX.*).<br>• If the data are all contained **within the manuscript and/or Supporting Information files**, enter the following: *All relevant data are within the manuscript and its Supporting Information files.*<br>• If neither of these applies but you are able to provide **details of access elsewhere**, with or without limitations, please do so. For example:<br><br>*Data cannot be shared publicly because of [XXX]. Data are available from the*	All the data and results are described fully and in detail in this manuscript.

XXX Institutional Data Access / Ethics Committee (contact via XXX) for researchers who meet the criteria for access to confidential data.<br><br>*The data underlying the results presented in the study are available from (include the name of the third party and contact information or URL).*<br>• This text is appropriate if the data are owned by a third party and authors do not have permission to share the data.<br><br>* typeset	
Additional data availability information:	

Powered by Editorial Manager® and ProduXion Manager® from Aries Systems Corporation

Manuscript

Click here to access/download;Manuscript;Manuscript.docx

Developing adapted wheat lines with broad-spectrum resistance against stem rust: Introgression of *Sr59* through backcrossing and selections based on genotyping-by-sequencing data

Mahboobeh Yazdani[1], Matthew N. Rouse[2,3], Brian J. Steffenson[3], Prabin Bajgain[4], Mehran Patpour[5] and Eva Johansson[1], Mahbubjon Rahmatov[1]

[1]Department of Plant Breeding, Swedish University of Agricultural Sciences, PO Box 101, SE-23053 Alnarp, Sweden; [2]United States Department of Agriculture, Agricultural Research Service, Cereal Disease Laboratory, St. Paul, MN 55108, USA; [3]Department of Plant Pathology, University of Minnesota, St. Paul MN 55108, USA; [4]Department of Agronomy and Plant Genetics, University of Minnesota, St. Paul, MN 55108; [5]Department of Agroecology, Aarhus University, Slagelse, Denmark

Email: Mahbubjon.Rahmatov@slu.se

Abstract

Wheat breeding against stem rust, caused by *Puccinia graminis* Pers. f.sp. *tritici* Eriks. and E. Henn. (*Pgt*), a highly devastating fungal disease of bread wheat (*Triticum aestivum* L.), is constantly challenged by the frequent emergence of new virulent *Pgt* races across wheat-growing continents, posing significant obstacles to resistance breeding in wheat. Utilizing combinations of broad-spectrum resistance genes would contribute to durable resistance against stem rust disease. The present study aimed at transferring a newly defined stem rust resistance gene, *Sr59* (from line TA5094). This line was developed through a *CSph1bM*-induced T2DS·2RL Robertsonian translocation conferring broad-spectrum resistance. However, due to the poor agronomic performance of line TA5094, it was necessary to eliminate the *CSph1bM* background to transfer *Sr59* into more adapted genetic backgrounds and assess its utility in wheat improvement. A total of 2070 BC_1F_1 and 1230 BC_2F_1 plants were derived from backcrossing of BAJ#1, KACHU#1, and REEDLING#1 with TA5094, as determined through combined stem rust seedling assays and molecular marker analyses. Genotyping-by-sequencing (GBS) results revealed the physical positions of 15,116 SNPs on chromosome 2R. Results showed that the adapted wheat genotypes used for backcrossing did not possess broad-spectrum resistance to the stem rust races tested, in contrast to line TA5094, which contains *Sr59* and shows resistance to all tested races. The

combined approach of stem rust seedling assays and kompetitive allele specific PCR (KASP) marker analysis proved successful in selecting and generating later generations, such as the BC_2F_2 population, that contained the *Sr59* gene - a finding also confirmed by GBS. In early-generation data from backcrossing, however, segregations diverged from 3:1, indicating that more than one gene may be responsible for the resistance reactions of *Sr59*. The use of GBS markers data (based on 40,584 SNPs across all seven wheat chromosomes) to transfer the recurrent parent background from the adapted cultivars to the later generation populations resulted in an average genome recovery of 71.2% in BAJ#1*2/TA5094, 69.8% in KACHU#1*2/TA5094, and 70.5% in REEDLING#1*2/TA5094 backcrossing populations. The GBS data verified a stable introgression of *Sr59* in the BC_2F_2 populations, evidenced by the presence of the *Ph1* locus and the absence of the 50,936,209 bp deletion, spanning from 396,630,846 bp to 447,567,055 bp, which is characteristic of the *CSph1bM* at the *Ph1* locus. Selection across generations up to BC_2F_9, using a combination of phenotypic selections, stem rust seedling assays, KASP markers, and GBS data, substantially accelerated the transfer of broad-spectrum resistance into adapted wheat genotypes, thus creating a form of speed breeding. In conclusion, this study demonstrates the successful introduction of the *Sr59* resistance gene into elite genetic backgrounds, providing a significant resource for wheat improvement to mitigate yield losses caused by stem rust.

Introduction

Wheat is globally an important source of calories and protein in the daily human diet [1]. Due to the rapid growth of the global population, an increase in wheat production of 60% has been projected as necessary in order to meet a similar food share for the human diet in 2050 [2]. Consequently, wheat yields need to be increased by at least 2% each year to meet this demand, a target that has currently not being reached attained [3]. The major constraints to achieving the needed yield increase are biotic and abiotic stresses that negatively affect crop performance, where in particular the three rust diseases have the potential to cause yield losses in severe outbreaks. Wheat. Among the biotic stresses, stem rust (caused by the fungus *Puccinia graminis* f. sp. *tritici* (*Pgt*)) is a major disease threat to wheat production across many regions of the world because it is capable of causing severe yield loss [4]. Stem rust can be

managed by fungicides applications, although this management strategy can be expensive, possibly environmentally destructive, and may cause and can be harmful to the environmental harmful and human health issues [5]. Hence, genetic resistance is the most economical and environmentally sustainable control measure to secure wheat yields from the threat of stem rust. The frequent emergence of new *Pgt* races is a challenge in breeding wheat for *Pgt* races challenges resistance. The emergence of the Ug99 race group is one such example as some races within the overcame all known and widely deployed stem rust (*Sr*) resistance genes, such as *Sr24*, *Sr31*, *Sr36*, and *SrTmp,* thereby causing great concern for widespread epidemics [6, 7].

Additionally, other widely virulent *Pgt* races such as TRTTF, TKTTF, TTRTF, TTKST, PRCTM and TTKTT have been detected with additional virulence combinations, including virulence to *Sr22+Sr24*, *Sr24+Sr31*, *Sr13b+Sr35+Sr37*, and *Sr24+Sr31+SrTmp* genes [8-10]. The emergence of these novel races and their spread into Europe is alarming since this disease has been largely absent for nearly 60 years [11-13]. Moreover, a high proportion of cultivars grown in Europe are susceptible to these emerging races, e.g. ~80% of wheat cultivars currently grown in the United Kingdom are susceptible to race TKTTF [11]. Resistance genes such as *Sr24*, *Sr31*, and *Sr38* present in German wheat cultivars contribute only are limited in their effectiveness against these novel races of *Pgt* [14]. Of the currently known and described wheat stem rust resistance (*Sr*) resistance genes, about 35 out of 73 were derived from the primary gene pool of wheat, and the majority of these do not confer broad-spectrum resistance [4]. *Sr31* resistance gene was, until recently, considered a highly effective and broad-spectrum resistance against all *Pgt* races as it conferred gene that was in many wheat cultivars over the past 30 years [7].

Rye (*Secale cereale* L., 2n = 14), belonging to the tertiary gene pool of wheat, is an important source of genes that can be used for bread wheat improvement against both abiotic and biotic stresses [15]. For instance, *Sr27*, *Sr31*, *Sr1RS*^{Amigo}, *SrSatu*, *Sr50*, and *Sr59* are important stem rust resistance genes that have been introduced into wheat from rye, and several of these genes have proven to confer broad-spectrum resistance [16-19]. However, the introgression of genes from wild relatives to wheat relies on meiotic recombination, which is complicated between rye and bread wheat. Hexaploid bread wheat (*Triticum aestivum* L., 2n = 6x = 42, AABBDD) was derived from *T. monococcum*/*T. urartu* (2n = 2x = 14, AA), *Aegilops sp.* (2n = 2x = 14, BB) and *Ae. tauschii* (2n = 2x = 14, DD) through spontaneous

interspecific crosses and allohexaploid wheat behaves as a diploid during meiosis [20]. This is due to the presence of *pairing homoeologous* (*Ph*) loci that strictly control pairing homology during meiosis. There are two major *Ph* loci; *Ph1* and *Ph2*, residing on chromosome 5BL and 3DS, respectively, that control homoeologous recombination in wheat [20, 21]. The deletion of the *Ph1* locus (*ph1b* in hexaploid and *ph1c* in tetraploid wheat) results in homoeologous recombination [22, 23]. As a result, a Chinese Spring line mutated at the *ph1b* locus *(CSph1bM)* has been used effectively to induce recombination between wheat and alien chromosomes. Thus, many broad spectrum resistance genes have been transferred using *CSph1bM*, including *Sr32* from *Ae. speltoides* [24], *Sr39* from *Ae. speltoides* [25], *Sr47* from *Ae. speltoides* [26], *Sr53* from *Ae. geniculata* [27], *Sr59* from *S. cereale* , *Sr43* from *Thinopyrum ponticum* [28], *Sr47* from *Ae. speltoides* [26], *Sr53* from *Ae. geniculata* [27], *Sr59* from *S. cereale* [16], and *Yr83* from *S. cereale* [29]. Also, *CSph1bM* has been used to transfer genes other than those for wheat resistance, e.g., end-use quality was improved by recombining the *Sec-1* (secalin) allele on the 1RS chromosome arm in wheat lines [30].

A large number of wheat-rye introgression lines were developed in the 1980s to 2000s by the late Professor Arnulf Merker at the Swedish University of Agricultural Sciences [31, 32]. Some of these wheat-rye substitution lines were used in field and greenhouse screenings to identify the line 'SLU238' [2R (2D) wheat-rye disomic substitution], which was characterized to confer broad-spectrum resistance to all *Pgt* races tested [33]. Thereafter, TA5094, a line derived from 'SLU238', was shown to possess a T2DS·2RL Robertsonian translocation with a stem rust resistance gene designated as *Sr59* [16]. Due to the lack of acceptable agronomic performance in TA5094 based on the *CSph1bM* background, there was an urgent need to transfer this gene to a more suitable genetic background and evaluate its potential use in wheat resistance breeding. The present study describes the transfer and subsequent evaluation of *Sr59* to agronomically suitable genetic backgrounds derived lines through: 1) marker-assisted backcross breeding; 2) stem rust seedling assessment; 3) background selection; and 4) physical mapping of the *Sr59* resistance gene on chromosome 2RL.

Materials and Method

Plant materials and stem rust seedling evaluations in parental lines

TA5094 was derived from a cross between *CSph1bM* and the line 'SLU238' [a 2R (2D) wheat-rye disomic substitution], and has been defined as a T2DS·2RL translocation containing the *Sr59* resistance gene [16]. Three wheat cultivars (BAJ#1, KACHU#1, and REEDLING#1), kindly provided by Ravi Singh (International Maize and Wheat Improvement Center (CIMMYT), El Batan, Mexico), were used as recurrent parents and crossed with TA5094 in the present study. When evaluating the progeny resulting offspring by seedling tests for stem rust reaction, molecular marker analysis kompetitive allele specific PCR [KASP] markers and genotyping by sequencing [GBS], the four lines of TA5094, *CSph1bM*, SLU238, and Chinese Spring (CSA) were used as controls. The above mentioned parental lines and controls were initially tested with the *Pgt* races TTTTF (isolate 01MN84A-1-2), TTTTF (Russia), QTHJC (C25; isolate 1541), TPMKC (C53; isolate 1373), RKQQC (C35; isolate 1312), RCRSC (isolate 77ND82A), TTRTF (Italy), TKTTF (13ETH60), TKTTF (Iraq and Sweden), TTKTT (isolate 14KEN58-1), TTKSK (isolate 04KEN156/04), TTKST (isolate 06KEN19v3), TTTSK (isolate 07KEN24-4), JRCQC (08ETH03-1), TRTTF (isolate 06YEM34-1), and LTBDC (Australian *Pgt* race 98-1,2,3,5,6).

Population development, stem rust seedling evaluations and molecular marker analysis

F_1 plants obtained from the crosses between line TA5094 and the recurrent parents (BAJ#1, KACHU#1, and REEDLING#1) were backcrossed to each of the corresponding recurrent parents, generating BC_1F_1 seeds. A total of 2,070 BC_1F_1 plants were assessed for their seedling responses to the *Pgt* race TTTTF (isolate 01MN84A-1-2) at the USDA-ARS Cereal Disease Laboratory and University of Minnesota, following the stem rust seedling assay previously described by [34], and [35]. For each recurrent parent, 94 resistant (i.e. in total 282 plants) BC_1F_1 plants were selected, which were then analyzed for the presence of *Sr59* by the use of three KASP markers, i.e. KASP_2RL_c25837C1, KASP_2RL_c21825C1 and KASP_2RL_c20194C2 [16]. Based on the KASP marker analysis, BC_1F_1 plants with *Sr59* were selected and used for backcrossing to produce BC_2F_1 plants. The backcross generated 1,230 BC_2F_1 plants that were assessed against *Pgt* race TTTTF. Resistant plants were selected and tested for the presence of *Sr59* with the three KASP markers. The selected plants carrying *Sr59* were selfed to produce the BC_2F_2 generation, which resulted in a total of 846 families (from all recurrent

parents). These families were again evaluated against the *Pgt* TTTTF and the resistant plants were selected and checked with the three KASP markers. The BC_2F_2 generation was also evaluated using race TTKSK, and the pattern of segregation was analyzed. From the BC_2F_2, additional generations (BC_2F_3, BC_2F_4, and BC_2F_5) were created through selfing and selection of twenty plants at each generation based on the seedling response to race TTTTF and presence of *Sr59* was validated by KASP markers. In addition, 10-15 BC_2F_4 and BC_2F_5 plants from each family were also tested for their seedling response to races TTKSK, TTTSK, and TRTTF. The BC_2F_5 families were also assessed against races TPMKC, QTHJC, RKQQC, and RCRSC. The segregation pattern data were assessed using a chi-square (χ^2) analysis.

Genotyping and Data analysis

The population was genotyped using genotyping by sequencing (GBS) as described by [36]. Tissue sampling and DNA extraction was carried as described in [50]. Approximately 10 cm of young leaf tissue from each of the donor parents (TA5094 and *CSph1bM*), recurrent parents, and a total of 128 of the selected BC_2F_2 plants (from all three recurrent parents) were collected in a 96-well tissue collection plate. Genomic DNA was isolated using the Qiagen BioSprint 96 instrument and the associated Qiagen BioSprint DNA Plant kit (https://www.qiagen.com/us/products/discovery-and-translational-research/dna-rna-purification/dna-purification/genomic-dna/biosprint-96-dna-plant-kit/#orderinginformation). DNA sequencing libraries were prepared and sequenced at the University of Minnesota Genomics Center. In brief, the isolated DNA was quantified with PicoGreen for GBS genotyping and normalized to 20 ng/µl. The GBS libraries were prepared in 96-plex using two restriction enzymes: --a rare cutter *Pst*I (5′-CTGCAG-3′), and a frequent cutter *Msp*I (5’-CCGG-3’) with a common reverse adapter ligated [36, 37]. Libraries were sequenced on Illumina HiSeq2500 (Illumina, San Diego, CA, USA). Obtained sequences in the FASTQ files were passed through a quality filter of Q > 30 and then de-multiplexed to obtain reads for each individual. Thereafter, the GBS reads were aligned to the International Wheat Genome Sequencing Consortium (IWGSC) Reference Sequence v1.0 (RefSeq v1.0) assembly and Rye Genome Sequencing Consortium Reference Sequence using the Burrow–Wheelers Alignment tool (BWA) v0.7.4 [38]. Marker discovery, i.e. identification of SNPs was

accomplished using Samtools+Bcftools [39]. SNPs with minor allele frequency (MAF) of less than 5% and more than 20% missing data were removed. After processing, 40,584 SNP markers for wheat and 15,116 SNP markers for chromosome 2R were retained for further analyses. Allele frequencies and genetic relationship between donor and recurrent parental lines were calculated using TASSEL v5.2.65 [40]. Principal components (PC) analysis was performed using the function 'prcomp' in R 4.0.2. MapChart 2.2 (https://www.wur.nl/en/show/mapchart.htm) was used to draw physical maps.

Selection of plants for the recovery of the recurrent parents

Progeny lines were selected based on their phenotypic and genomic similarity to the recurrent parents and were subsequently used in the next backcross generation. The phenotypic parameters of each backcross generation (BC_1F_1 and BC_2F_1) were evaluated in the greenhouse to determine whether the plants were similar to the recurring parents based on their height, tillering, heading date, flowering, spike characteristics (with or without awns), seed fertility, and maturity day. Following that, in the BC_2F_2 generation, background selection for alleles similar to those of the recurrent parents was performed using GBS data distributed across all 42 wheat and the 2R rye chromosomes. After this, individual plants from each generation (BC_2F_3 to BC_2F_6) were carefully selected based on their highest phenotypic and genotypic similarities to the recurrent parents, ensuring a consistent inheritance of desired traits across generations.

The BC_2F_7 and BC_2F_8 lines, resistant to TTTTF and TTKSK races, were sown in the field for phenotyping evaluations. In one replication field evaluation, these lines were sown in small plots to assess phenotypic traits similar to the recurrent parents. Data was collected regarding characteristics such as the number of days to 50% flowering and maturity, plant height, tillering, lodging, susceptibility to diseases (e.g., rusts, powdery mildew, septoria, FHB, etc.), and the grain color.

Results

Stem rust seedling response in the parental lines

Seventeen *Pgt* races were used to evaluate the stem rust seedling response of parental lines and revealed that SLU238 and TA5094 were broadly resistant, exhibiting infection types (ITs) of ;1 to 1+2- to all

races in this experiment (Table 1). The recurrent parents (BAJ#1, KACHU#1, and REEDLING#1) were found susceptible to several of the *Pgt* races and exhibited ITs of 3+4 to races TTTTF (USA and Russia), TTKTT, TTRTF, TTKSK, TTTSK, and TRTTF (Table 1). For races RKQQC, RCRSC, TKTTF (Sweden, Iraq, Ethiopia), LTBDC (Australian *Pgt* race 98-1,2,3,5,6), QTHJC, and TPMKC, recurrent parents had ITs ranging from 0 to 11+ (Table 1). CSph1bM and CSA were highly susceptible to all seventeen *Pgt*16 of 17 races that were tested, indicating that no resistance genes were present in these two lines (Table 1). Based on the virulence profile of the different *Pgt* races, REEDLING#1, BAJ#1, and KACHU#1 were postulated to carry resistance genes *Sr11* and *Sr38* (Table 1).

Stem rust seedling evaluations and marker-assisted backcrossing

The evaluation of the BC_1F_1 and BC_2F_1 populations using race TTTTF suggested the presence of a major resistance gene following crossbreeding with BAJ#1 (Table 2). The P-values of all other BC_1F_1 and BC_2F_1 populations resulting from these crosses were <0.05, indicating a segregation pattern deviating from the expected 1:1 ratio. Such deviation suggests potential segregation distortion or the involvement of more than one major gene in resistance. However, the segregation ratio was close to 3:1 (P>0.05) for the BC_2F_2 populations from the backcrosses to BAJ#1 and REEDLING#1 (Table 2), indicating a single dominant major resistance gene. To verify the presence of *Sr59* in plants that were selected for further generations, a number of resistant plants were selected and genotyped with three KASP markers (Table 2) from each of the BC_1F_1, BC_2F_1 and BC_2F_2 populations, and some of the plants containing the gene were transplanted for backcrossing and selfing (Table 2). The BC_2F_2 populations were also phenotyped with race TTKSK, which resulted in a significant deviation from the expected 3:1 segregation ratio (P<0.01). Instead, the segregation ratio was closer to 14:2 (P>0.05), suggesting the presence of a major gene along with one or more additional genes co-acting with the major gene. The BC_2F_4 and BC_2F_5 populations, obtained through assessments against race TTTTF, and subjecting selected resistant plants to KASP genotyping, showed a similar high level of resistance (IT ;1- to ;11+) against races TTTSK, TTKST, and TRTTF as their resistant parental lines (Table 1), thereby indicating successful transfer of *Sr59* to the later generations. The BC_2F_5 families also displayed the same responses (IT ;01- to ;11+) as SLU238 and TA5094 when evaluated against races TPMKC, QTHJC, RKQQC, and RCRSC.

GBS genotyping and physical location of *Sr59* in the 2R chromosome

Through the alignment of raw GBS reads against the rye line 'Lo7' (International Rye Genome Sequencing Consortium (IRGSC)) reference genome, followed by filtering to remove SNPs with missing values ≤20% and minor allele frequency (MAF) ≥ 5%, the physical positions of the 15,116 SNPs obtained were mapped to chromosome 2R at 347694 bp to 945773747 bp (Figure 1). The physical position of the 15,116 SNPs from GBS (rye alleles) are shown in red color, whereas blue color indicates the presence of wheat alleles when mapping the BC_2F_2 populations (Figure 2b). The physical positions of three KASP markers (c20194_115, c25837_157, and c21825_230) and the GBS showed that *Sr59* was located in the 2RL segment. The three KASP markers were also used to track the *Sr59* introgression into the recurrent parent's background through the crossing scheme utilized in the present study. BLASTN searches of the three KASP markers against IRGSC, positioned the *Sr59* resistance gene between 914812226 bp and 943109279 bp on the 2RL (Figure 1). The presence of the 2RL was also clearly verified in lines SLU238, SLU239, and TA5094, as demonstrated by the red color, whereas the 2BL (wheat) was verified in BAJ#1, KACHU#1, REEDLING#1, CSA, *CSph1bM*, and susceptible lines (6-BAJ-S, 23-KACHU-S, 33-Reed-S, 35-Reed-S, and 45-Reed-S) as demonstrated by the blue color (Figure 2b). A strong association was observed between the presence of the rye/wheat alleles determined by the GBS dataset and the resistance (red color)/susceptible (green color) reactions to race TTTTF and TTKSK (Figure 2a-c). Most of the BC_2F_3 lines showed homozygosity (100% red color) for resistance towards the TTTTF race, although some lines segregated (40 percent red and 60 percent green; Figure 2a). The reaction towards TTKSK was tested in the BC_2F_3 lines, where the homozygous lines showed IT ;1 (100% red color), while the segregating lines showed ;1 to 3+4 (varying percentages of green and red color; Figure 2c). The BC_2F_3 lines are shown as 100% green color for both races (TTTTF and TTKSK; Figure 2a,c). A PCA analysis based on the results of the GBS data (15,116 SNPs on chromosome 2R) clustered the genotypes evaluated into five distinct clusters: 1) SLU238; 2) TA5094 and 24 BC_2F_2 populations; 3) BC_2F_3 lines from these populations; 4) BAJ#1, KACHU#1, REEDLING#1; and 5) *CSph1bM* and CSA (Figure 3).

Recovery of the recurrent parent (background selection)

To select lines that resembled their recurrent parents as much as possible (with the exception of the addition of *Sr59*), a total of 40,584 SNPs across all 21 chromosomes were used to identify the most suitable lines in the BC_2F_2. Based on polymorphic SNPs, whole-wheat genome PCA identified five distinct clusters: 1) *CSph1bM* and CSA; 2) SLU238 and TA5094; 3) BC_2F_2 BAJ#1 population; 4) BC_2F_2 KACHU#1 population; and 5) BC_2F_2 REEDLING#1 population (Figure 4). The PCA showed that most of the BC_2F_2 of a recurrent parent clustered at the same plot, indicating genomic recovery of the recurrent parent genome. As shown in Table 3, the percentage of genome recovery for the recurrent parents ranged from 66% to 75% across the three BC_2F_2 populations. The plants with the highest recurrent parent genome recovery and carrying *Sr59* were selected to generate homozygous lines through selfing.

Phenotypic selection in the greenhouse and field conditions

Besides using SNPs to produce lines resembling recurrent parental lines, selection was carried out in greenhouse and field evaluations with plant height and awns/awnless spikes being key phenotypic characters considered during crossing and backcrossing as these characters differed between SLU238 and TA5094 versus the recurrent parents, BAJ#1, KACHU#1, and REEDLING#1 (Table 2). Thus, forty BC_1F_1 plants with maximum phenotypic similarity to the recurrent parents were used for developing the BC_2F_1 population. In BC_2F_2 to BC_2F_6 populations, plants were selected to four characters, i.e. plant height, awns/awnless, days to maturity, and seed fertility. Then, BC_2F_7 lines showing a homozygous resistance reaction towards race TTTTF and positive KASP marker data were sown in the field during 2020. The following phenotypic parameters were considered for selecting single plants in the field in 2020: plant stands, tillering, plant height, awns/awnless spikes, lodging, days to maturity, and seed fertility. Following the phenotypic analysis described above, BC_2F_8 populations were sown in the greenhouse and selfed to produce another generation, and BC_2F_9 populations were planted in the field during the spring of 2021 to select plants whose phenotypic similarity to the recurrent parents was the greatest. The three KASP markers (c20194_115, c25837_157, and c21825_230) were used again to verify the presence of the *Sr59* resistance gene in the individual BC_2F_7 to BC_2F_9 plants.

_Ph1_ allele status

A BLAST search against the IWGSC wheat reference sequence v1.0, resulted in 2,050 SNPs from the GBS were annotated in the range of 16637 bp to 712890017 bp on chromosome 5B (Figure 5). Furthermore, a deletion breakpoint of 50936209 bp (51 Mb), located from 396630846 bp to 447567055 bp, was detected in the *CSph1bM*, indicating the place of the *Ph1* locus on the 5B chromosome (Figure 5). GBS also showed the presence of the *Ph1b* deletion in TA5094, and two BC_2F_2 populations (31-Kachu and 34-Kachu), whereas it was not detected in the other BC_2F_2 populations, SLU238, CSA, or the recurrent parents. These results show that most of the BC_2F_2 populations carry the *Ph1* allele, and that the status of the 2DS.2RL (and thus the introgression of *Sr59* into the wheat genetic background of the recurrent parents) is stable.

Discussion

In this study, the *Sr59* stem rust resistance gene was transferred, utilizing TA5094 as a donor of rye chromatin, into three elite wheat lines through marker-assisted backcrossing selection and stem rust seedling screening (BC_1F_1 to BC_2F_5). The stem rust seedling screening and KASP marker analysis allowed us to trace the presence/absence of *Sr59* from the parental lines to all of the progeny through all generations. The use of novel genomic tools, such as GBS, facilitated intense selection pressure that increased the probability of recovering the elite recurrent parent background genome while at the same time preserving the translocation fragment 2DS.2RL containing *Sr59*. The GBS background selection accurately identified both the translocated 2DS.2RL and the deletion region on 5BL (*ph1b* deletion). *Sr59* contributed stable resistance as demonstrated by seedling screening against multiple *Pgt* races and KASP marker analysis across all developed populations.

Here, we present a GBS and marker assisted method to eliminate the *ph1b* deletion while transferring the *Sr59* into the genetic background of three widely adapted wheat cultivars (BAJ#1, KACHU#1, and REEDLING#1) from CIMMYT through backcrossing to ultimately produce wheat lines suitable for breeders. Initially, the *Sr59* resistance gene was selected based on the stem rust seedling response and marker-assisted backcrossing selection. Previous studies have shown that two backcrossing generations

may recover about 87.5% of the recurrent parent genome [41]. The use of a large backcrossing population is a common practice to introgress resistance genes from an alien genome into an elite background [25]. Aside from marker-assisted backcrossing, we used GBS genotyping to select plants with the greatest amount of the recurrent parent genome. A recurrent parent genome recovery of 71.2%, 69.8%, and 70.5% was observed in the BC_2F_2 populations. This fairly low recovery might be the result of the lack of 2DL in the GBS dataset, due to the fact that we started off with a 2DS.2RL translocation in the transferring parent. In GBS genotyping, the A and B genomes are reported to have the highest number of SNPs, while the D genome has the lowest number [42]. Evolutionary history and gene flow may be the reason for the poor D genome representation [43]. GBS has in previous studies been found to be both inexpensive and robust for genotyping of crop genomes, as it enables the discovery of a high number of genome-wide markers, often SNPs [37]. Several studies have demonstrated that GBS detects small introgressions in wheat and barley [44, 45]. The present study aligned GBS reads and located the physical positions of SNPs in wheat and 2R rye chromosomes based on the reference genome RefSeq v1.0 and the International Rye Genome Sequencing Consortium. In this alignment, GBS demonstrated the physical positions of 40,584 SNPs across the wheat genome and 15,116 SNPs for the 2R chromosome. Through this high-throughput genotyping procedure, it was possible to detect both translocation lines and non-translocation lines in the BC_2F_2 populations.

We also used principal component (PC) analysis to visualize the grouping of lines based on their genetic relationship, i.e. based on the differences of 2RL segments transferred to the BC_2F_2 progeny (Figure 3). Basically, the susceptible BC_2F_2 plants and parental lines without the 2R chromosome (CSph1bM, BAJ#1, KACHU#1, REEDLING#1) clustered with a positive PC1 (Figure 3). Likewise, 24 BC_2F_2 resistant plants grouped closely to the lines TA5094 and SLU238, indicating the presence of the whole 2RL chromosome segment (Figure 3). Additionally, a total of 28 BC_2F_2 resistant plants clustered differently, indicating that these lines most likely resemble each other in regards to their genomic composition, for both wheat or rye genome segments (Figure 3, B). Some factors, such as chromosomal segment rearrangements, segmental duplications, and differences in recombination frequencies caused by genomic structural variations, may explain the marker orders observed in this short segment. The

introgression of rye chromosomes into wheat genomes can result in structural changes and rearrangements, as the heterochromatin DNA of rye chromosomes can interfere with chromosome synapsis [2, 51]. There may have been chromosome rearrangements in the BC_2F_2 population that resulted in a shorter 2RL segment than in the other 24 BC_2F_2 lines. This study showed that the *Sr59* resistance gene in the 2RL chromosome segment is stable for normal transmission through the male gamete, preventing segregation distortion in cultivar development. No segregation distortion was observed in any of the three populations evaluated, neither for the TTTTF race, nor for the TTKSK race. However, previous studies have shown that segregation distortion is a common feature when alien chromosomes are introgressed in the wheat genome [25]. Creating chromosome-specific SNP markers covering target chromosomes and facilitating homologous recombination on chromosomes containing resistance genes can assist in tracking rye resistance genes within wheat more effectively by minimizing chromosome transfer and reducing the likelihood of linkage to undesirable alleles. Line SLU238 wheat-rye disomic substitution carrying 2R (2D) chromosome exhibited effective resistance to several virulent races of stem rust, which was used to develop 2DS.2RL wheat-rye translocation lines [16]. Substitution lines serve as bridging materials in the development of wheat-alien translocation lines [25]. By incorporating distinct alien chromosome segments with desired traits through chromosome translocations, linkage drag can be reduced [25, 28].

For the three KASP markers (c20194_115, c25837_157, and c21825_230), BLASTN was used and their positions were mapped at 914812229 bp to 943109279 (28 Mb), which physically mapped in previous study [16]. Based on GBS reads, we observed a deletion spanning from 396630846 Mb to 447567055 Mb on chromosome 5B in TA5094 and two BC_2F_2 plants (31-Kachu and 34-Kachu), indicating the deletion of the CSph1bM allele. The deletion on the 5B chromosome was not detected in the other BC_2F_2 plants, all carrying the *Ph1* allele, preventing true homology in pairing. New *ph1b* deletion-specific markers based on 90K SNPs have been developed to accurately identify the *ph1b* deletion region [46]. A successful cross-over between wheat chromosome and its wild relatives is challenging, which is why CSph1bM mutants are recommended for inducing meiotic homoeologous recombination [23]. Several resistance genes, such as *Sr32*, *Sr47*, *Sr39*, *Sr59*, and *Yr83* were successfully transferred using CSph1bM

mutants [16, 24, 25, 29]. This approach provides an effective means of introducing beneficial traits from wild relatives for wheat improvement. The *Sr11* and *Sr38* resistance genes were postulated to be present in three recurrent parents from CIMMYT (Table 1). The *Sr11* and *Sr38* resistance genes have previously been reported as widely prevalent in wheat cultivars worldwide [47, 48], the recurrent parents may carry both genes. Due to the limited agronomic performance of the CSph1bM mutant and the elimination of the *ph1b* allele in line TA5094, it is necessary to transfer *Sr59* into adapted cultivars. Here, we successfully transferred the *Sr59* from TA5094 to the genetic background of the three cultivars BAJ#1, KACHU#1, and REEDLING#1 was, and the progenies showed proven resistance to TTTTF (USA), TKTTF (Ethiopia), QTHJC, TPMKC, RKQQC, RCRSC, TTKST, TTKSK, TTTSK, TRTTF, and LTBDC (Australian Pgt race 98-1,2,3,5,6). Following backcrossing, morphological and agronomic characteristics with the greatest similarity to the recurrent parents were considered. Other resistance genes such as *SrTA1662*, *Yr15*, and *Sr39* have been transferred in previous work using backcrossing to recover the recurrent parent phenotype [25, 41, 49]. In conclusion, *Sr59* offers a broad-spectrum resistance to stem rust races, making it a valuable gene for wheat improvement. The reliability of KASP markers for *Sr59* also makes them suitable for marker-assisted selection of stem rust resistance in wheat breeding. The present study facilitates for further production of stem rust-resistant wheat cultivars, developed with the *Sr59* resistance in elite background. The new lines in the adapted background provide additional assets for improving wheat yields and preventing stem rust losses.

Acknowledgments

We thank the University of Minnesota Genomics Center for its genotyping services and the University of Minnesota Supercomputing Institute for providing computational resources. We also gratefully acknowledge the financial support the Swedish Research Council Vetenskapsrådet and FORMAS provided, which enabled this research.

Author Contributions

MR and MNR conceptualized and designed the study. MR was responsible for developing crosses, genotyping, phenotyping, and analyzing the data. MY conducted the KASP marker analysis in BC_2F_7

to BC_2F_9 and helped format the manuscript. PB was responsible for managing the analysis of the GBS dataset. MNR, BJS, and MP contributed to the stem rust seedling resistance work and assisted with manuscript editing. MR and EJ wrote the initial draft of the manuscript, and all authors contributed to its final editing and revision.

References

1. [illegible]hewry PR, Hey SJ. The contribution of wheat to human diet and health. Food Energy Secur. 2015;4(3):178-202. doi: 10.1002/fes3.64. PubMed PMID: WOS:000367088200002.
2. Appels R, Eversole K, Stein N, Feuillet C, Keller B, Rogers J, et al. Shifting the limits in wheat research and breeding using a fully annotated reference genome. Science. 2018;361(6403):eaar7191. doi: 10.1126/science.aar7191.
3. Cassman KG, Grassini P. A global perspective on sustainable intensification research. Nat Sustain. 2020;3(4):262-8. doi: 10.1038/s41893-020-0507-8. PubMed PMID: WOS:000526392900005.
4. Singh RP, Hodson DP, Jin Y, Lagudah ES, Ayliffe MA, Bhavani S, et al. Emergence and Spread of New Races of Wheat Stem Rust Fungus: Continued Threat to Food Security and Prospects of Genetic Control. Phytopathology. 2015;105(7):872-84. doi: 10.1094/Phyto-01-15-0030-Fi. PubMed PMID: WOS:000360927100004.
5. Oliver RP. A reassessment of the risk of rust fungi developing resistance to fungicides. Pest Manag Sci. 2014;70(11):1641-5. doi: 10.1002/ps.3767. PubMed PMID: WOS:000342850800001.
6. Patpour M, Hovmoller MS, Justesen AF, Newcomb M, Olivera P, Jin Y, et al. Emergence of Virulence to SrTmp in the Ug99 Race Group of Wheat Stem Rust, Puccinia graminis f. sp tritici, in Africa. Plant Dis. 2016;100(2):522-. doi: 10.1094/Pdis-06-15-0668-Pdn. PubMed PMID: WOS:000370305300054.
7. Pretorius ZA, Singh RP, Wagoire WW, Payne TS. Detection of Virulence to Wheat Stem Rust Resistance Gene Sr31 in Puccinia graminis. f. sp. tritici in Uganda. Plant Dis. 2000;84(2):203. Epub 2000/02/01. doi: 10.1094/PDIS.2000.84.2.203B. PubMed PMID: 30841334.
8. Olivera P, Newcomb M, Szabo LJ, Rouse M, Johnson J, Gale S, et al. Phenotypic and Genotypic Characterization of Race TKTTF of Puccinia graminis f. sp tritici that Caused a Wheat Stem Rust Epidemic in Southern Ethiopia in 2013-14. Phytopathology. 2015;105(7):917-28. doi: 10.1094/Phyto-11-14-0302-Fi. PubMed PMID: WOS:000360927100008.
9. Olivera PD, Sikharulidze Z, Dumbadze R, Szabo LJ, Newcomb M, Natsarishvili K, et al. Presence of a Sexual Population of Puccinia graminis f. sp. tritici in Georgia Provides a Hotspot for Genotypic and Phenotypic Diversity. Phytopathology. 2019;109(12):2152-60. doi: 10.1094/Phyto-06-19-0186-R. PubMed PMID: WOS:000502011800017.
10. Hovmøller MS, Patpour, M., Rodriguez-Algaba, J., Thach, T., Justesen, A.F., and Hansen, J.G. . GRRC report of yellow and stem rust genotyping and race analyses 2020. Aarhus University, Flakkebjerg, DK-4200 Slagelse, Denmark. https://agro.au.dk/fileadmin/www.grcc.au.dk/International_Services/Pathotype_YR_results/GRRC_annual_report_2020.pdf, 2021.
11. Lewis CM, Persoons A, Bebber DP, Kigathi RN, Maintz J, Findlay K, et al. Potential for re-emergence of wheat stem rust in the United Kingdom. Commun Biol. 2018;1. doi: ARTN 13 10.1038/s42003-018-0013-y. PubMed PMID: WOS:000461126500013.
12. Firpo PDO, Newcomb M, Flath K, Sommerfeldt-Impe N, Szabo LJ, Carter M, et al. Characterization of Puccinia graminis f. sp tritici isolates derived from an unusual wheat stem rust outbreak in Germany in 2013. Plant Pathol. 2017;66(8):1258-66. doi: 10.1111/ppa.12674. PubMed PMID: WOS:000410746600005.
13. Bhattacharya S. Deadly new wheat disease threatens Europe's crops. Nature. 2017;542(7640):145-+. doi: 10.1038/nature.2017.21424. PubMed PMID: WOS:000393737500005.

14. Flath K, Miedaner T, Olivera PD, Rouse MN, Jin Y. Genes for wheat stem rust resistance postulated in German cultivars and their efficacy in seedling and adult-plant field tests. Plant Breeding. 2018;137(3):301-12. doi: 10.1111/pbr.12591. PubMed PMID: WOS:000434231800007.
15. Johansson E, Henriksson T, Prieto-Linde ML, Andersson S, Ashraf R, Rahmatov M. Diverse Wheat-Alien Introgression Lines as a Basis for Durable Resistance and Quality Characteristics in Bread Wheat. Frontiers in Plant Science. 2020;11. doi: ARTN 1067
10.3389/fpls.2020.01067. PubMed PMID: WOS:000560098900001.
16. Rahmatov M, Rouse MN, Nirmala J, Danilova T, Friebe B, Steffenson BJ, et al. A new 2DS.2RL Robertsonian translocation transfers stem rust resistance gene Sr59 into wheat. Theor Appl Genet. 2016;129(7):1383-92. Epub 2016/03/31. doi: 10.1007/s00122-016-2710-6. PubMed PMID: 27025509.
17. The TT, Gupta RB, Dyck PL, Appels R, Hohmann U, Mcintosh RA. Characterization of Stem Rust Resistant Derivatives of Wheat Cultivar Amigo. Euphytica. 1991;58(3):245-52. doi: Doi 10.1007/Bf00025256. PubMed PMID: WOS:A1991HZ63400007.
18. Marais GF, Marais AS. The Derivation of Compensating Translocations Involving Homoeologous Group-3 Chromosomes of Wheat and Rye. Euphytica. 1994;79(1-2):75-80. doi: Doi 10.1007/Bf00023578. PubMed PMID: WOS:A1994PZ62500010.
19. Mago R, Zhang P, Vautrin S, Simkova H, Bansal U, Luo MC, et al. The wheat Sr50 gene reveals rich diversity at a cereal disease resistance locus. Nat Plants. 2015;1(12). doi: Artn 15186
10.1038/Nplants.2015.186. PubMed PMID: WOS:000365704700005.
20. Riley R, Chapman V. Genetic Control of the Cytologically Diploid Behaviour of Hexaploid Wheat. Nature. 1958;182(4637):713-5. doi: DOI 10.1038/182713a0. PubMed PMID: WOS:A1958ZQ50000023.
21. Mellosampayo T. Genetic Regulation of Meiotic Chromosome Pairing by Chromosome-3d of Triticum-Aestivum. Nature-New Biol. 1971;230(9):22-+. PubMed PMID: WOS:A1971I753500015.
22. Giorgi B, Barbera F. Increase of homoeologous pairing in hybrids between a ph mutant of T. turgidum L. var. durum and two tetraploid species of Aegilops: *Aegilops kotschyi* and *Ae. cylindrica*. Cereal Res Commun. 1981;9(205-2).
23. Sears ER. An induced mutant with homoeologous pairing in wheat. Can J Genet Cytol. 1977;19:585–93.
24. Mago R, Verlin D, Zhang P, Bansal U, Bariana H, Jin Y, et al. Development of wheat-Aegilops speltoides recombinants and simple PCR-based markers for Sr32 and a new stem rust resistance gene on the 2S#1 chromosome. Theor Appl Genet. 2013;126(12):2943-55. Epub 2013/08/31. doi: 10.1007/s00122-013-2184-8. PubMed PMID: 23989672.
25. Niu Z, Klindworth DL, Friesen TL, Chao S, Jin Y, Cai X, et al. Targeted introgression of a wheat stem rust resistance gene by DNA marker-assisted chromosome engineering. Genetics. 2011;187(4):1011-21. Epub 2011/01/19. doi: 10.1534/genetics.110.123588. PubMed PMID: 21242535; PubMed Central PMCID: PMCPMC3070511.
26. Klindworth DL, Niu Z, Chao S, Friesen TL, Jin Y, Faris JD, et al. Introgression and characterization of a goatgrass gene for a high level of resistance to ug99 stem rust in tetraploid wheat. G3 (Bethesda). 2012;2(6):665-73. Epub 2012/06/13. doi: 10.1534/g3.112.002386. PubMed PMID: 22690376; PubMed Central PMCID: PMCPMC3362296.
27. Liu W, Rouse M, Friebe B, Jin Y, Gill B, Pumphrey MO. Discovery and molecular mapping of a new gene conferring resistance to stem rust, Sr53, derived from Aegilops geniculata and characterization of spontaneous translocation stocks with reduced alien chromatin. Chromosome Res. 2011;19(5):669-82. Epub 2011/07/06. doi: 10.1007/s10577-011-9226-3. PubMed PMID: 21728140.
28. Niu Z, Klindworth DL, Yu G, T LF, Chao S, Jin Y, et al. Development and characterization of wheat lines carrying stem rust resistance gene Sr43 derived from Thinopyrum ponticum. Theor Appl Genet. 2014;127(4):969-80. Epub 2014/02/08. doi: 10.1007/s00122-014-2272-4. PubMed PMID: 24504553.
29. Li J, Dundas I, Dong C, Li G, Trethowan R, Yang Z, et al. Identification and characterization of a new stripe rust resistance gene Yr83 on rye chromosome 6R in wheat. Theor Appl Genet. 2020;133(4):1095-107. Epub 2020/01/20. doi: 10.1007/s00122-020-03534-y. PubMed PMID: 31955232.

30. Lukaszewski AJ. Manipulation of the 1RS.1BL translocation in wheat by induced homoeologous recombination. Crop Sci. 2000;40(1):216-25. doi: DOI 10.2135/cropsci2000.401216x. PubMed PMID: WOS:000085505300035.

31. Merker A. The Rye Genome in Wheat Breeding. Hereditas. 1984;100(2):183-91. doi: DOI 10.1111/j.1601-5223.1984.tb00118.x. PubMed PMID: WOS:A1984SV71000040.

32. Forsstrom PO, Merker A. Sources of wheat powdery mildew resistance from wheat-rye and wheat-Leymus hybrids. Hereditas. 2001;134(2):115-9. doi: DOI 10.1111/j.1601-5223.2001.00115.x. PubMed PMID: WOS:000172203900003.

33. Rahmatov M, Rouse MN, Steffenson BJ, Andersson SC, Wanyera R, Pretorius ZA, et al. Sources of Stem Rust Resistance in Wheat-Alien Introgression Lines. Plant Dis. 2016;100(6):1101-9. doi: 10.1094/Pdis-12-15-1448-Re. PubMed PMID: WOS:000375634200009.

34. Rouse MN, Wanyera R, Njau P, Jin Y. Sources of Resistance to Stem Rust Race Ug99 in Spring Wheat Germplasm. Plant Dis. 2011;95(6):762-6. doi: 10.1094/Pdis-12-10-0940. PubMed PMID: WOS:000290879700020.

35. Stakman EC, Stewart, D.M., and Loegering W.Q. Identification of physiologic races of Puccinia graminis var. tritici. United States Department of Agriculture, Agricultural Research Service, Beltsville, MD E-617. 1962.

36. Elshire RJ, Glaubitz JC, Sun Q, Poland JA, Kawamoto K, Buckler ES, et al. A robust, simple genotyping-by-sequencing (GBS) approach for high diversity species. PloS one. 2011;6(5):e19379.

37. Poland JA, Brown PJ, Sorrells ME, Jannink J-L. Development of high-density genetic maps for barley and wheat using a novel two-enzyme genotyping-by-sequencing approach. PloS one. 2012;7(2):e32253.

38. Li H, Durbin R. Fast and accurate short read alignment with Burrows–Wheeler transform. bioinformatics. 2009;25(14):1754-60.

39. Li H. A statistical framework for SNP calling, mutation discovery, association mapping and population genetical parameter estimation from sequencing data. Bioinformatics. 2011;27(21):2987-93.

40. Bradbury PJ, Zhang Z, Kroon DE, Casstevens TM, Ramdoss Y, Buckler ES. TASSEL: software for association mapping of complex traits in diverse samples. Bioinformatics. 2007;23(19):2633-5.

41. Randhawa HS, Mutti JS, Kidwell K, Morris CF, Chen X, Gill KS. Rapid and targeted introgression of genes into popular wheat cultivars using marker-assisted background selection. PloS one. 2009;4(6):e5752.

42. Bajgain P, Rouse MN, Anderson JA. Comparing Genotyping-by-Sequencing and Single Nucleotide Polymorphism Chip Genotyping for Quantitative Trait Loci Mapping in Wheat. Crop Sci. 2016;56(1):232-48. doi: 10.2135/cropsci2015.06.0389. PubMed PMID: WOS:000368266100024.

43. Przewieslik-Allen AM, Wilkinson PA, Burridge AJ, Winfield MO, Dai XY, Beaumont M, et al. The role of gene flow and chromosomal instability in shaping the bread wheat genome. Nat Plants. 2021;7(2). doi: 10.1038/s41477-020-00845-2. PubMed PMID: WOS:000613614100001.

44. Tiwari VK, Wang S, Sehgal S, Vrána J, Friebe B, Kubaláková M, et al. SNP discovery for mapping alien introgressions in wheat. BMC genomics. 2014;15(1):1-11.

45. Keilwagen J, Lehnert H, Berner T, Beier S, Scholz U, Himmelbach A, et al. Detecting Large Chromosomal Modifications Using Short Read Data From Genotyping-by-Sequencing. Frontiers in Plant Science. 2019;10(1133). doi: 10.3389/fpls.2019.01133.

46. Gyawali Y, Zhang W, Chao S, Xu S, Cai X. Delimitation of wheat ph1b deletion and development of ph1b-specific DNA markers. Theoretical and Applied Genetics. 2019;132(1):195-204.

47. Bajgain P, Rouse MN, Bulli P, Bhavani S, Gordon T, Wanyera R, et al. Association mapping of North American spring wheat breeding germplasm reveals loci conferring resistance to Ug99 and other African stem rust races. BMC Plant Biol. 2015;15:249. Epub 20151014. doi: 10.1186/s12870-015-0628-9. PubMed PMID: 26467989; PubMed Central PMCID: PMCPMC4606553.

48. Helguera M, Khan IA, Kolmer J, Lijavetzky D, Zhong-qi L, Dubcovsky J. PCR assays for the Lr37-Yr17-Sr38 cluster of rust resistance genes and their use to develop isogenic hard red spring wheat lines. Crop Sci. 2003;43(5):1839-47. doi: DOI 10.2135/cropsci2003.1839. PubMed PMID: WOS:000185174300036.

49. Olson EL, Rouse MN, Pumphrey MO, Bowden RL, Gill BS, Poland JA. Simultaneous transfer, introgression, and genomic localization of genes for resistance to stem rust race TTKSK (Ug99) from Aegilops tauschii to wheat. Theoretical and Applied Genetics. 2013;126(5):1179-88.
50. Ashraf R, Johansson E, Vallenback P, Steffenson BJ, Bajgain P, Rahmatov M. Identification of a Small Translocation from 6R Possessing Stripe Rust Resistance to Wheat. Plant Dis. 2023 Mar;107(3):720-729. doi: 10.1094/PDIS-07-22-1666-RE. PMID: 35900348.
51. Thomas JB, Kaltsikes PJ. A possible effect of heterochromatin on chromosome pairing. Proc. Natl. Acad. Sci. USA. 1974 71, 2787–2790

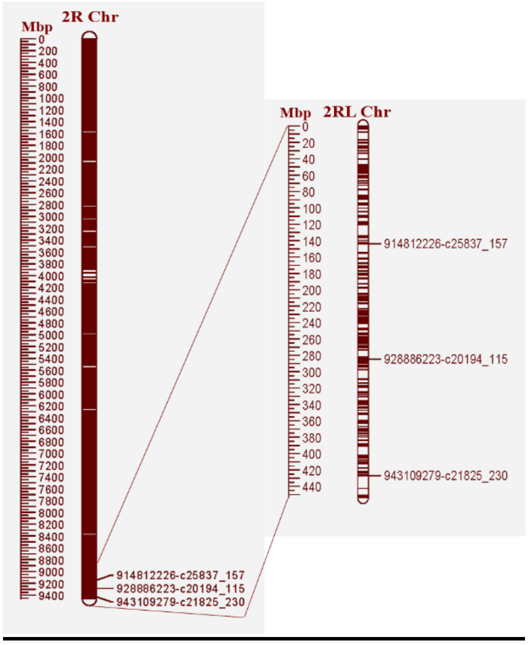

Figure 1. The physical position of *Sr59*

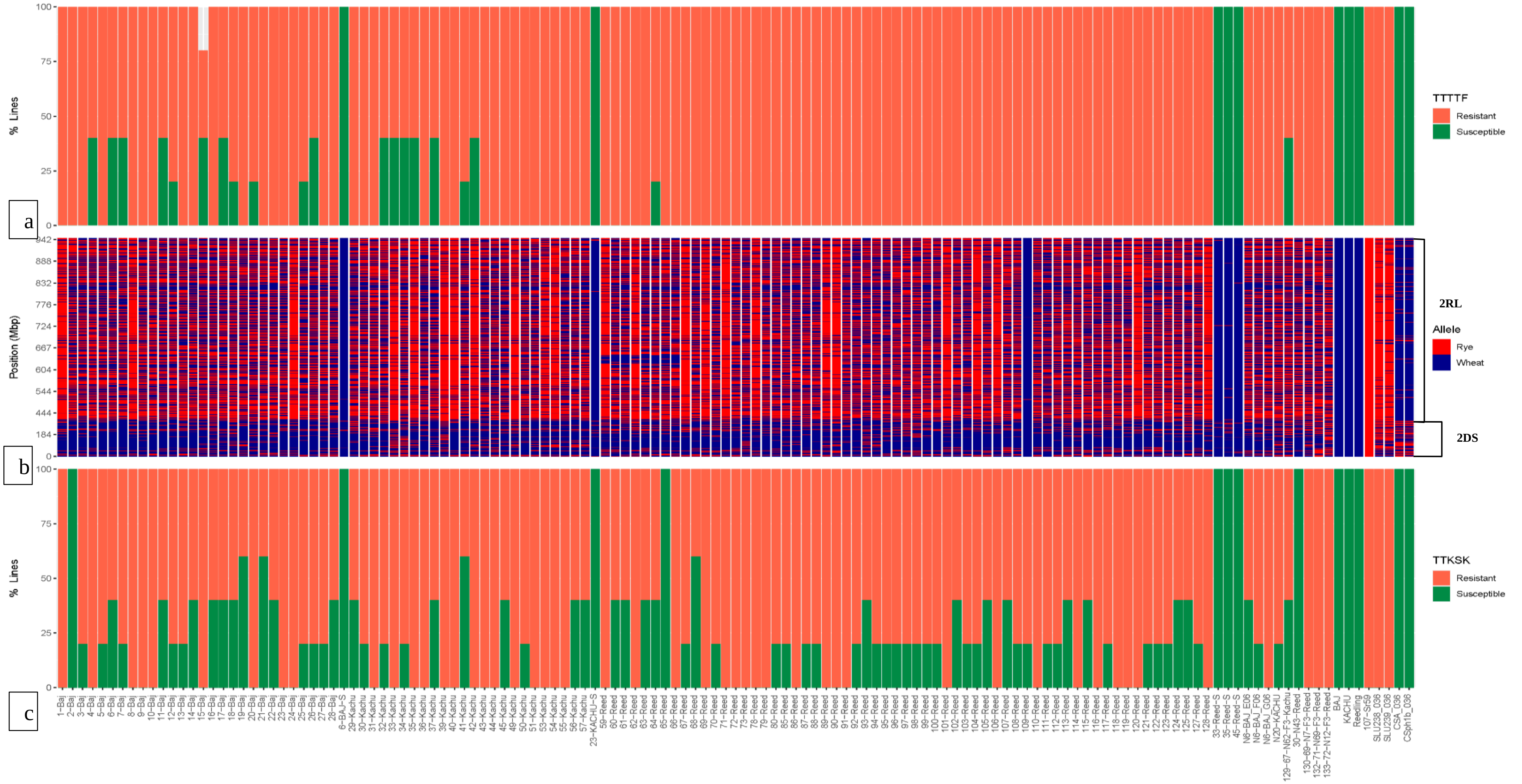

a
b
c
% Lines
Position (Mbp)
TTTTF
Resistant
Susceptible
TTKSK
Resistant
Susceptible
Allele
Rye
Wheat
2RL
2DS

Figure 2. Physical position of the 2DS.2RL wheat-rye translocation region. Blue color represents the proportion of wheat allele and red color represent proportion of rye allele in the BC_2F_2 population. The results are based on 15,116 SNPs on the chromosome 2R – A) BC_2F_3 B) BC_2F_2, and C) BC_2F_3.

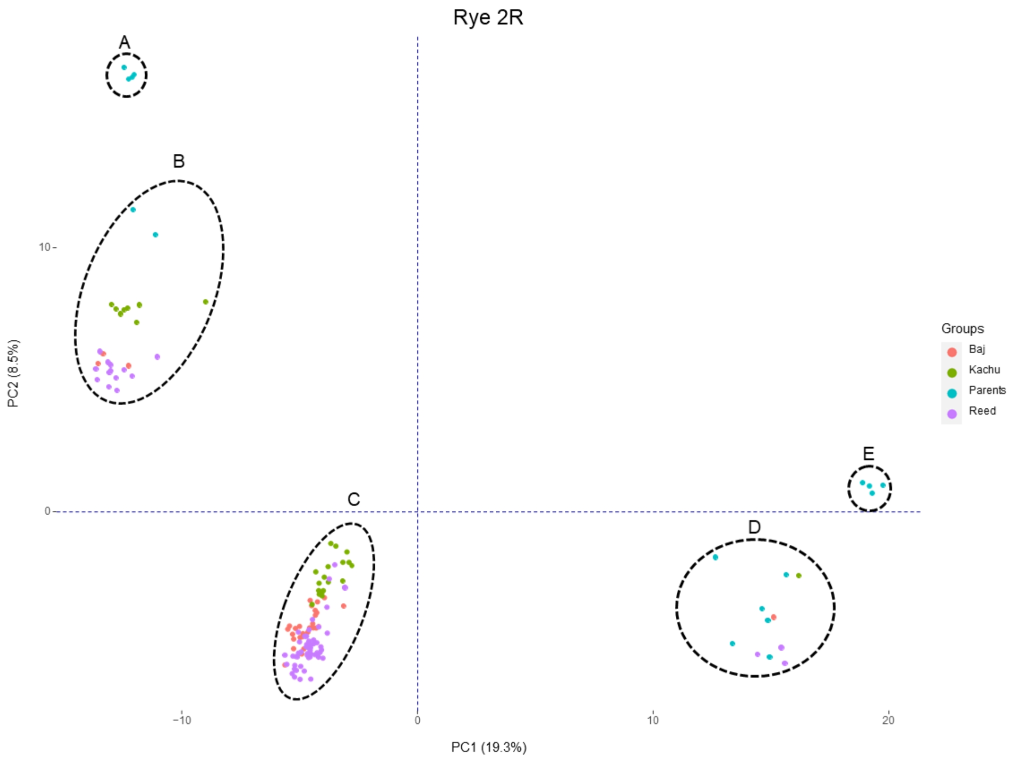

Figure 3. Relationship among the lines based on polymorphic sites in the rye 2R chromosome as obtained from Genetic by Sequence (GBS) method

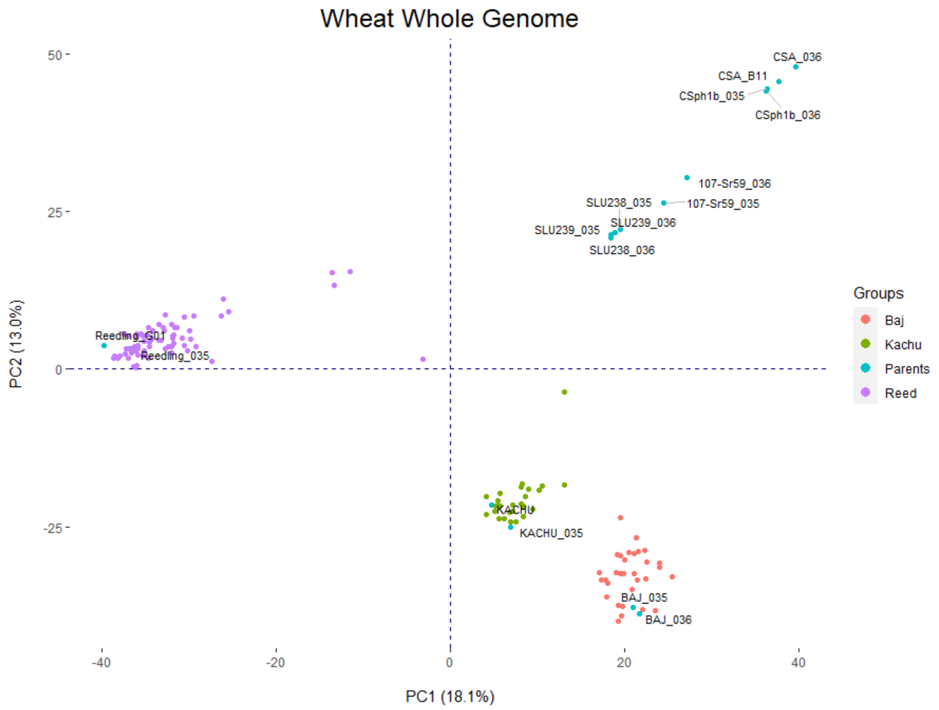

Figure 4. Relationship among the lines based on polymorphic sites in the entire wheat genome obtained from Genetic by Sequence (GBS) method.

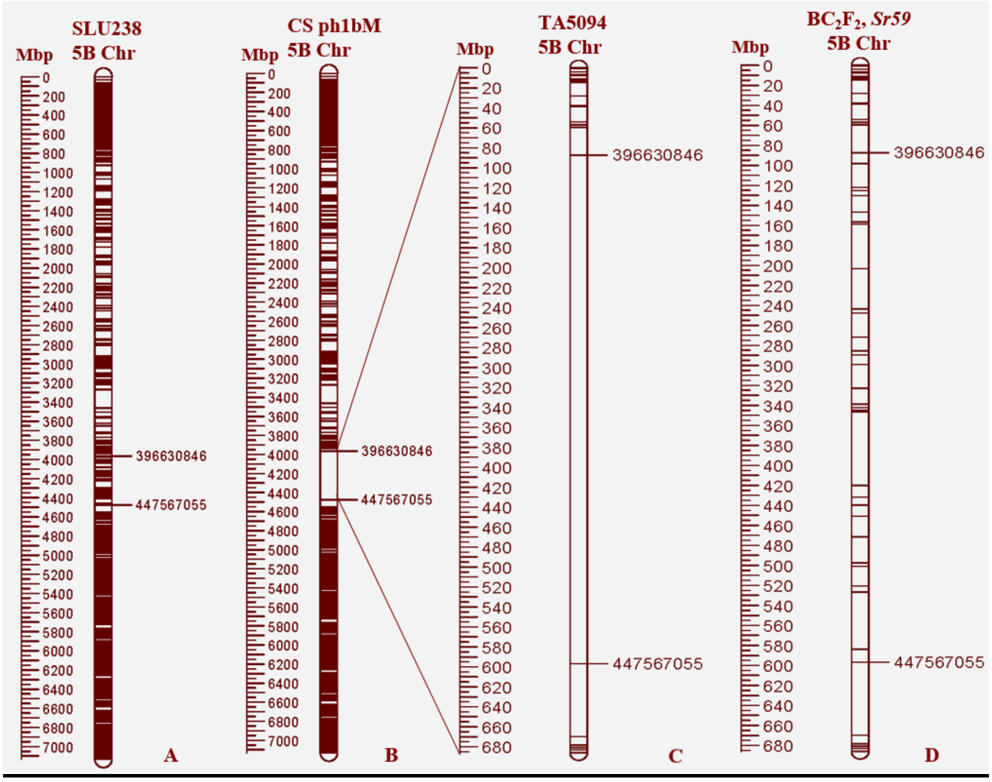

Figure 5. The status of Ph1 allele (Ph1 deletion) in different lines evaluated in this study

Table 1. Stem rust seedling response in the parental lines

Parental lines	TTTTF USA	TTRTF Italy	TTTTF Russia	TKTTF +*Sr25* Iraq	TTKTT Rwanda	QTHJC USA	TPMKC USA	RKQQC USA	RCRSC USA	TKTTF Sweden	TKTTF Ethiopia	TTKSK Kenya	TTTSK Kenya	TTKST Kenya	JRCQC Ethiopia	TRTTF Yemen	LTBDC Australia	Gene postulation	Spike phenotype
CSph1bM	4	4	4	4	4	4	3+	4	4	4	4	4	4	4	4	4	4	None	Awnless
CSA	4	4	4	4	4	4	3+	4	4	4	4	4	4	4	4	4	4	None	Awnless
SLU238	1+2-	;1	11+	11+	;1	11-	11+	11+	;11-	11+	11+	;1-	;1-	;1-	11+	;11+	11+	*Sr59*	Awnless
TA5094	1+2-	;1	11+	1+	;1	11-	11+	11+	11+	11+	11+	;1-	;1-	;1-	11+	;11+	11+	*Sr59*	Awnless
BAJ#1	4	4	4	;1-	4	;0	11+	;0	11-	;1-	11+	4	4	4	N.A.	4	22-	*Sr11+Sr38*	Awn
KACHU#1	4	4	4	;1-	3+	11+	11-	;0	;1-	;1-	;1-	4	4	4	N.A.	4	11+	*Sr11+Sr38*	Awn
REEDLING#1	4	4	4	;1-	4	;0	11-	;0	;1-	;1-	11+	4	4	4	N.A.	4	11+	*Sr11+Sr38*	Awn

Note: N.A. – Note available

Table 2. Crossing and backcrossing, and selection procedures[illegible]h *Pgt* race TTTTF and KASP markers in BC_1F_1 and BC_2F_1

Cross	Generation	*Pgt* race TTTTF Resistant	*Pgt* race TTTTF Susceptible	χ2	P-value	No. of plants for KASP analysis	No. of transplanted plants	Selected plants for GBS	Expected phenotypes for selection
BAJ#1*1/TA5094	BC_1F_1	265	195	10.65	0.001	94	40	-	Awn and short height*
BAJ#1*2/TA5094	BC_2F_1	135	155	1.37	0.24	94	40	-	Awn and short height
	BC_2F_2	180	66	0.65	>0.1	40	32	32	Awn and short height
KACHU#1*1/TA5094	BC_1F_1	380	310	7.1	0.007	94	40	-	Awn and short height
KACHU#1*2/TA5094	BC_2F_1	235	195	4.35	0.036	94	40	-	Awn and short height
	BC_2F_2	215	35	24.2	<0.001	40	30	30	Awn and short height
REEDLING#1*1/TA5094	BC_1F_1	520	400	15.65	<0.001	94	40	-	Awn and short height
REEDLING#1*2/TA5094	BC_2F_1	278	232	4.14	0.041	94	40	-	Awn and short height
	BC_2F_2	432	127	2.32	>0.1	80	66	66	Awn and short height

Note: * The selection based on height ranging from 95 to 100 cm.

Table 3. Recurrent parent genome recovery in BC_2F_2 generation using 40,584 genome-wide SNPs

Cross	Generation	Genome recovered			Genome expected
		Minimum	Maximum	Average	
BAJ#1*2/TA5094	BC_2F_2	66.00%	74.60%	71.20%	87.50%
KACHU#1*2/TA5094	BC_2F_2	63.40%	74.20%	69.80%	87.50%
REEDLING#1*2/TA5094	BC_2F_2	66.2%	73.40%	70.50%	87.50%
