# Supplementary material for: Developing adapted wheat lines with broad-spectrum resistance to stem rust: Introgression of Sr59 through backcrossing and selections based on genotyping-by-sequencing data
Source: PLoS One. 2023 Oct 12;18(10):e0292724. doi: 10.1371/journal.pone.0292724 (PMC10569509; doi:10.1371/journal.pone.0292724)
Supplement: Attachment — Submitted filename: Response to Reviewers.docx [file pone.0292724.s002.docx]

**Response to Reviewers**

**Journal Requirements:**

1. **Please ensure that your manuscript meets PLOS ONE's style requirements, including those for file naming. The PLOS ONE style templates can be found at provided links.**

Thank you for your suggestions and comments. We have adhered to the guidelines and styles provided by PLOS ONE at the given links.

2. **We suggest you thoroughly copyedit your manuscript for language usage, spelling, and grammar. If you do not know anyone who can help you do this, you may wish to consider employing a professional scientific editing service.**

The manuscript has been reviewed and edited by a professional language editor in the UK. Hopefully, this revision will now comply with PLOS ONE's language standards.

Language editor: Mary McAfee. Email: [mary.scantext@btinternet.com](mailto:mary.scantext@btinternet.com)

3. **Thank you for stating the following financial disclosure: "For this research financial support obtained from the Swedish Research Council Vetenskapsrådet and FORMAS"**

There was no involvement of the funding agencies in the study's design, the collection and analysis of data, the decision to publish, or the preparation of the manuscript. We will write according to your suggestion. Thanks for your suggestions.

**Review Comments to the Author**

**Reviewer #1**

**Suggestion by the Reviewer 1:** The findings reported in this manuscript, along with the material that will be available, is valuable to the wheat research community in providing broad stem rust resistance due to the *Sr59* allele that has been transferred into more adapted germplasm. Overall, the science is sound in this manuscript and ready for publication. Throughout, there are a few comments on some grammar mistakes, clarification on figures/tables, and also one little part of the materials and methods. Otherwise, the manuscript is really clear to read, and I congratulate the authors on doing a wonderful job on their research.

**Response:** Thanks for your positive suggestions and comments.

**Suggestion by the Reviewer 1: Major comments:**
The abstract is too long. The limit listed for the journal is 300 words and the current abstract is over 400. I suggest finding a way to only include the most relevant findings for most people who will use these wheat lines. The information on the Ph1 locus could be eliminated from the abstract to reduce word count.

**Response:** We have revised the Abstract according to your recommendations and have now limited it to 298 words. Thank you for your suggestion.

**Suggestion by the Reviewer 1:** What was the field location used for phenotypic evaluations? Please provide a description, along with coordinates. Part of this research was done in Sweden, Denmark, and the US, which would greatly change the results for this part of the project. Also, please provide a more detailed description of small plots, such as rows, length, type, etc. Also, why was only one replication in the field used?

**Response:** The phenotypic evaluations took place in Svalöv, Sweden, and we have provided detailed descriptions of the field trials. Due to seed limitations and the use of BC_2_F_7_ and BC_2_F_8_ lines, where we noticed some segregation in certain families, we opted for a single replication. Seedling resistance screenings against stem rust races were performed at the Global Rust Reference Center at Aarhus University in Denmark, as well as the University of Minnesota and the United States Department of Agriculture's Agricultural Research Service Cereal Disease Laboratory in Minnesota, USA.

**Suggestion by the Reviewer 1:** For all the figures and tables, not enough information is given for each. Please expand with more details for each one.

**Response:** Thanks for your suggestion. We have revised the descriptions of the figures and tables in order to make them more informative.

**Suggestion by the Reviewer 1:** For figure 1: which reference/genome are the physical positions based on, what are the dark red sections, and state that the markers are the three KASP markers.

**Response:** Here, we utilized the genome assembly of the rye-inbred line 'Lo7' described by Rabanus-Wallace et al., 2021. We have also updated the figure's description.

**Suggestion by the Reviewer 1:** For figure 2: the panel description of a, b, and c doesn’t make what is in the figure so please correct, give a description of the colors used in the figure legend/text section, and more details on this figure.

**Response:** We have revised the descriptions of the figures in order to make it more informative.

**Suggestion by the Reviewer 1:** For figure 3, please describe each cluster A-E and make sure it matches what was in the text of the manuscript.

**Response:** Thank you for pointing it out. We have detailed the clusters A-E and verified that their descriptions are consistent with the text.

**Suggestion by the Reviewer 1:** For figure 4, please include cluster information.

**Response:** This change was incorporated in the manuscript revision.

**Suggestion by the Reviewer 1:** For figure 5, what is the reference used for all the physical positions?

**Response:** Here, we utilized the Wheat Genome Sequencing Consortium Reference Sequence v1.0 (RefSeq v1.0) for assembly. In the case of rye

**Suggestion by the Reviewer 1:** For table 1: what is the scale used or the citation for this, please remove the “e” from the end of “note” (I think you meant to say “not available”).

**Response:** We removed this accordingly

**Suggestion by the Reviewer 1: Minor comments:**
Line 48: Include scientific name for wheat.

**Response:** *Triticum aestivum* L. added in the text

**Suggestion by the Reviewer 1:** Line 52: Either take out “reached” or “attained”.

**Response:** Accordingly deleted

**Suggestion by the Reviewer 1:** Line 54: Random “Wheat.” as its own sentence.

**Response:** Wheat deleted

**Suggestion by the Reviewer 1:** Line 58-59: This sentence doesn’t flow smoothly. It may sound better to rewrite as “…and may cause human health issues and be harmful to the environment [5].”

**Response:** Here is a revised version of the sentence: Although fungicide application can effectively manage stem rust, it is associated with drawbacks such as high costs, significant environmental impact, and negative effects on human health

**Suggestion by the Reviewer 1:** Line 62: remove “within” and rewrite sentence so has corrected grammar.

**Response:** Here is a revised version of the sentence: An example of this is emergence of the Ug99 race group, which is capable of overcoming all known and extensively deployed stem rust (*Sr*) resistance genes, including *Sr24*, *Sr31*, *Sr36*, and *SrTmp*. This constant adaptation of the pathogen has increased concerns about global epidemics. Current line is 65 to 68.

**Suggestion by the Reviewer 1:** Line 71-72: Please fix grammar mistakes in this sentence as well.

**Response:** Here is a revised version of the sentence: Resistance genes such as *Sr24*, *Sr31*, and *Sr38*, present in German wheat cultivars, are limited in their effectiveness against these novel races of *Pgt*. Current line is 75 to 76.

**Suggestion by the Reviewer 1:** Line 73: remove “about” before 35. Otherwise, state “about half”.

**Response:** Accordingly removed

**Suggestion by the Reviewer 1:** Line 115: Please state what type of cultivars these are. The thought behind this suggestion is so readers who are interested in requesting these lines can know what growth type (pretty sure all three are spring) and wheat species (*Triticum aestivum*).

**Response:** Yes, they are all spring bread wheat. In the text “spring bread” is added.

**Suggestion by the Reviewer 1:**Line 234: Please state which figure these KASP markers are on (pretty sure it is figure 1). The authors had switched briefly to discussing Figure 2b so it is confusing which one the KASP are on.

**Response:** Thank you for bringing this issue to our attention. Figure 1 has been added

**Suggestion by the Reviewer 1:** Lines 249-251: For figure 3, the clusters are labelled as letters in the figure, which don’t match the numbers in the text. Please change either the text or the figure labelling so they match.

**Response:** We have updated the text and figure to reflect this change.

**Suggestion by the Reviewer 1:** Line 256-258 discusses clusters in Figure 4, but no clusters are labelled in this figure. Please label this figure so it matches the text.

**Response:** Accordingly, the cluster was placed as shown in the figure

**Suggestion by the Reviewer 1:** Lines 312-315: Table 3 should be referenced in this section.

**Response:** Table 3 is added as a reference to this section. Current line is 305 to 307.

**Suggestion by the Reviewer 1:** Line 334: There is no Figure 3B as stated in the text. Please correct to either figure 3 or the correct figure.

**Response:** The B is accordingly removed

**Suggestion by the Reviewer 1:** Line 372: Remove “was” after REEDLING#1.

**Response:** Accordingly removed

**Reviewer #2**

**Suggestion by the Reviewer 2:** TA5094 is the T2DS•2RL translocation line with the stem rust resistance gene *Sr59*. The present study described the transfer and subsequent evaluation of *Sr59* into agronomically better genetic backgrounds using wheat cultivars as backcross parents. This is a necessary step for wheat breeding.

**Response:** Thanks for your positive suggestions and comments.

**Suggestion by the Reviewer 2:** Some suggestions have been placed in the attached file.

**Response:** Thank you for your suggestions on the manuscript. All of your suggestions have been appropriately incorporated into the manuscript.

**Suggestion by the Reviewer 2:** A major concern is how to physically map the *Sr59* resistance gene on chromosome 2RL (Figure 1). The authors should present a more detailed description.

**Response:** We have revised the description of Figure 1, and we hope it now better explains *Sr59*'s physical mapping.

**Suggestion by the Reviewer 2:** How to draw the conclusion according to the P-value in the BC1F1 and BC2F1.

**Response:** We calculated the P-value to assess the statistical significance of the observed impact of *Sr59* on the TTTTF race, and here we draw conclusions based on this analysis.
